# Supplementary material for: HDAC3 restrains CD8-lineage genes to maintain a bi-potential state in CD4+CD8+ thymocytes for CD4-lineage commitment
Source: eLife. 2019 Jan 18;8:e43821. doi: 10.7554/eLife.43821 (PMC6338460; doi:10.7554/eLife.43821)
Supplement: Supplementary file 1. [file elife-43821-supp1.docx]

**Supplementary Table 1.** Primers used for ChIP-seq and qChIP.

| **ChIP-seq Primer** | **Primer Sequence** |
| --- | --- |
| Intergenic-F | CCTGCTGCCTTGTCTCTCTC |
| Intergenic-R | ATGGCCTAGGGATTCCAGCA |
| PABPC1-TSS-F | ATCCCACAGCTTGTGGCGGG |
| PABPC1-TSS-R | TCTCGCCATCGGTCGCTCTC |
| T1-TSS-F | GAGACGCCGATCCGCCGAAG |
| T1-TSS-R | ACTCTCCACTCCCACGCGCT |
| Actb-TSS-F | CCCGGCAAGCCGAATAGGCA |
| Actb-TSS-R | ACCAGACGCTACGATCACGCC |

| **qChIP Primer** | **Primer Sequence** | **Figure** |
| --- | --- | --- |
| Runx3-SE-F | CAACCACAGAACCACAAGGC | Figure 7A, 7D |
| Runx3-SE-R | CAAAGGGCCACCTCATCCTC | Figure 7A, 7D |
| Runx3-promoter-F | GAGCCCCTTCCCACCATTTA | Figure 7A, 7D |
| Runx3-promoter-R | GAATGAACGAGGCTCACCC | Figure 7A, 7D |
| Patz1-F | CAAGCCTCCAGTTGGGCTAT | Figure 7A, 7D |
| Patz1-R | GGGTCCTCGGAGATGGGTAA | Figure 7A, 7D |
| IL21r-prom-F | GTGTTTCAGTCGCACACAGC | Figure 5H |
| IL21r-prom-R | TCAGACAAGCAAGTGACCGT | Figure 5H |
